# Parosteal Lipoma

**DOI:** 10.5334/jbsr.3502

**Published:** 2024-02-19

**Authors:** Adel Arfi, Arnaud Devriendt

**Affiliations:** 1 Department of Radiology, UZ Brussels, VUB, Brussels, Belgium; 2 Department of Radiology, CHU Brugmann, Brussels, Belgium

**Keywords:** Parosteal lipoma, soft tissue mass, juxtacortical focal ossification, radiography, magnetic resonance imaging

## Abstract

*Teaching Point:* Recognizing the distinct imaging features of parosteal lipoma.

## Case History

A 71-year-old male treated in rheumatology for ankylosing spondylitis presented with swelling in the right distal forearm. No history of trauma or relevant medical, familial history was reported. A physical examination revealed a firm, immobile swelling in the right distal forearm without scars or dilated veins.

**Figure 1:**
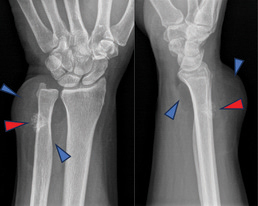
X-ray of the wrist face and profile.

The X-ray revealed a radiolucent soft-tissue mass at the distal of the forearm in direct connection with the distal ulna (Figure 1, blue arrowheads), accompanied by an irregular osseous protuberance on the underlying bone (Figure 1, red arrowheads).

**Figure 2:**
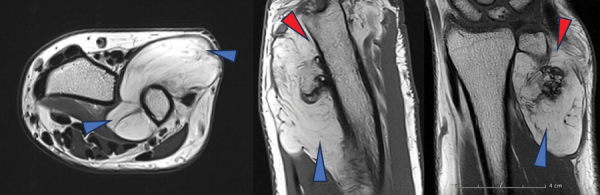
Axial T2-weighted image - Sagittal and Coronal T1-weighted image.

**Figure 3:**
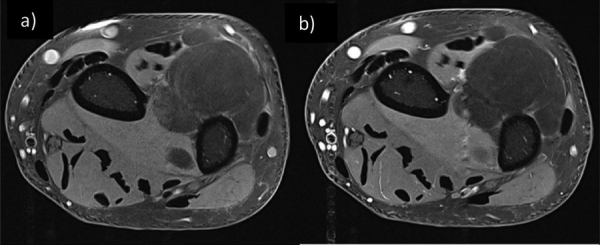
Axial T1-weighted image with fat saturation before (a) and after intravenous gadolinium injection (b).

Magnetic resonance imaging (MRI) further detailed a well-circumscribed, multi-lobulated mass in direct relation to the posterolateral distal ulnar meta-diaphysis (Figure 2, blue arrowheads). The mass displayed T1 and T2 hypersignals and exhibited a loss of signal on fat suppression (FS), containing thin septa without suspicious enhancement (Figure 3 (a) T1 FS without contrast and (b) T1 FS with contrast). Juxtacortical focal ossification was present without continuity with the medullary space of the underlying bone (Figure 2, red arrowheads).

The findings were typical for a parosteal lipoma.

A discussion regarding resection and/or biopsy was proposed to the patient, but he declined any invasive procedure and opted for clinical follow-up.

## Comments

Parosteal lipoma represents a highly uncommon lipomatous lesion, accounting for a mere 0.3% of all lipomas. Predominantly localized in the thigh, forearm, calf, and arm, this lesion typically manifests in individuals aged between 40 and 60, although it can occur across a broad age spectrum [1]. Characterized as a benign neoplastic process, parosteal lipoma lacks reported instances of degeneration.

Patients commonly present with an asymptomatic soft tissue mass. Depending on the lesion's size and location, local compression of structures, notably muscles and nerves, may induce symptoms such as nerve palsy [1].

Radiographically, parosteal lipomas exhibit a distinctive appearance. They present as well-circumscribed radiolucent masses in proximity to bone, accompanied by variable osseous alterations at the attachment site. These alterations encompass hyperostosis (the most frequently observed), bone deformity, and cortical erosion.

MRI of parosteal lipomas reveals a homogenous, lobulated lipomatous mass adherent to the cortical surface of the adjoining bone. Osseous excrescences, when present, can be distinguished from osteochondromas by their lack of continuity with the marrow space of the underlying bone and the absence of a cartilaginous cap. Within the lesion, low-intensity strands with moderate enhancement may be discerned, corresponding to fibro-vascular strands commonly identified in lipomatous lesions.

Computed tomography (CT) also depicts both components effectively, providing a particularly detailed view of the osseous structure.

For symptomatic patients, the recommended course of action is complete surgical resection, while incidental findings may warrant a conservative approach with abstention.
